# A Pancancer Analysis of the Expression Landscape and Clinical Relevance of Fibroblast Growth Factor Receptor 2 in Human Cancers

**DOI:** 10.3389/fonc.2021.644854

**Published:** 2021-04-21

**Authors:** Juanni Li, Kuan Hu, Jinzhou Huang, Lei Zhou, Yuanliang Yan, Zhijie Xu

**Affiliations:** ^1^Department of Pathology, Xiangya Hospital, Central South University, Changsha, China; ^2^Department of Hepatobiliary Surgery, Xiangya Hospital, Central South University, Changsha, China; ^3^Department of Oncology, Mayo Clinic, Rochester, MN, United States; ^4^Department of Anesthesiology, Third Xiangya Hospital of Central South University, Changsha, China; ^5^Department of Pharmacy, Xiangya Hospital, Central South University, Changsha, China; ^6^National Clinical Research Center for Geriatric Disorders, Xiangya Hospital, Central South University, Changsha, China

**Keywords:** *FGFR2*, gene fusion, gene alteration, prognosis, pancancer

## Abstract

**Background:** Fibroblast growth factor receptor 2 (FGFR2) is frequently altered in tumors and one of the top therapeutic targets in cholangiocarcinoma (CHOL) with *FGFR2* fusions. Although there have been several studies on individual tumors, a comprehensive analysis of *FGFR2* genetic aberrations and their simultaneous clinical implications across different tumors have not been reported.

**Methods:** In this study, we used the large comprehensive datasets available, covering over 10,000 tumor samples across more than 30 cancer types, to analyze *FGFR2* abnormal expression, methylation, alteration (mutations/fusions and amplification/deletion), and their clinical associations.

**Results:** Alteration frequency, mutation location distribution, oncogenic effects, and therapeutic implications varied among different cancers. The overall mutation rate of *FGFR2* is low in pancancer. CHOL had the highest mutation frequency, and fusion accounted for the major proportion. All these fusion aberrations in CHOL were targetable, and an FDA-approved drug was approved recently. Uterine corpus endometrial carcinoma (UCEC) had the highest number of *FGFR2* mutations, and the most frequently mutated positions were S252W and N549K, where the functional impact was oncogenic, but targeted therapy was less effective. Additionally, DNA methylation was associated with *FGFR2* expression in several cancers. Moreover, FGFG2 expression and genetic aberrations showed clinical associations with patient survival in several cancers, indicating their potential for application as new tumor markers and therapeutic targets.

**Conclusions:** This study showed the full *FGFR2* alteration spectrum and provided a broad molecular perspective of *FGFR2* in a comprehensive manner, suggesting some new directions for clinical targeted therapy of cancers.

## Introduction

The fibroblast growth factor receptor 2 (FGFR2) gene is located at chromosome 10q26 and encodes two major isoforms, FGFR2b and FGFR2c, which act as FGF receptors with different functional domains and ligand specificities (1–4). The FGFR2 protein belongs to a superfamily of four membrane-bound receptor tyrosine kinases (RTKs) (5–8), which includes FGFR1, FGFR2, FGFR3, and FGFR4 and exhibits biological activity by interacting with fibroblast growth factor (FGF) ligands (9). FGFs contain at least 22 members and are involved in numerous critical functions, including development, cell growth, differentiation, and survival (10, 11).

FGFR2 exhibits oncogenic and anti-oncogenic roles in a context-dependent manner (12–14). Various *FGFR2* alterations have been reported in multiple cancers. Enhanced FGFR2 signaling, mediated by *FGFR2* alterations containing genetic amplification, mutation, and fusion, has been observed in several cancers and is associated with tumorigenesis (6). Frequent activating mutations of *FGFR2* are discovered in 10% of bladder urothelial carcinomas (BLCAs) and UCECs. Additionally, *FGFR2* fusion aberrations are commonly observed in CHOL (15–17).

Owing to its important role in tumorigenesis, *FGFR2* has recently been considered a critical therapeutic target for cancer (18–22). FGFR inhibitors are classified into several classes, such as pan-FGFR-targeted tyrosine kinase inhibitors (TKIs), multitarget TKIs, and FGFR2 monoclonal antibodies (23). Currently, multiple FGFR2 inhibitors have been investigated in preclinical and clinical studies. Especially in CHOL, pemigatinib, a pan-FGFR-targeted inhibitor, was approved in the USA for the targeted therapy of patients (24, 25). Other FGFR2 inhibitors are also undergoing clinical development for use in CHOL or other solid tumors, such as BGJ398, Debio 1347, and erdafitinib (26–28).

Previous studies on FGFR2 in tumors are limited to individual tumor types and/or to limited sample sizes. In this analysis, we fill this gap in a comprehensive manner by taking advantage of the large datasets from TCGA. We systematically profiled abnormal *FGFR2* expression, methylation, alteration, and clinical implications across 32 TCGA cancer types covering over 10,000 tumor samples. We investigated changes in and links between *FGFR2* mRNA and methylation status across TCGA cancers. Then, we explored *FGFR2* alteration patterns, mutation patterns, and their functional impact and clinical therapeutic implications in distinct cancers and investigated *FGFR2* copy number variant (CNV) patterns and their impact on gene expression. Finally, survival association analysis was conducted to investigate the aberration patterns and potential clinical significance of *FGFR2* across different cancer types. In general, these findings highlight the critical role of *FGFR2* in oncogenesis and provide a potential therapeutic target for cancers.

## Materials and Methods

### Data Acquisition and Reanalysis Using Different Bioinformatics Tools

The Gene Expression Profiling Interactive Analysis 2 (GEPIA2) database was used to compare *FGFR2* mRNA expression profiles between tumors and normal tissues. The TCGA and GTEx gene expression data of *FGFR2* were downloaded and recomputed from raw RNA-Seq data by the UCSC Xena project (http://xena.ucsc.edu/) based on a standard pipeline (29) to make data from these two sources more compatible (30). They consulted with medical experts to determine the most appropriate sample grouping for tumor-normal comparisons. The datasets were stored in a MySQL relational database (version 5.7.17). Next, we analyzed differential methylation of *FGFR2* and downstream genes between tumors and their paired normal tissues across various TCGA cancer types by using GSCALite platform (31). After logging into the GSCALite website, we used the “TCGA Cancer-Methylation” module and chose 32 TCGA cancer types for analyzing differential methylation of *FGFR2* and downstream genes between tumors and normal tissues. In the final results figure generated by the GSCALite, the methylation profiles of *FGFR2* and its downstream genes were shown in only 14 cancer types. Then, the correlation between methylation and the expression of *FGFR2* and downstream genes in different cancers was further been explored by the same platform. GSCALite is a bioinformatics platform for gene set cancer analysis, and offers several analysis types such as methylation analysis, drug sensitivity for gene analysis, genomic variations and their survival analysis, and so on (31). The data for *FGFR2* fusion gene were downloaded from the TCGA Fusion Gene Database (32), which cover fusion genes predicted by PRADA analysis of RNA sequencing data across 32 TCGA cancer types.

The cBioportal database contains large-scale cancer genomics data and provides an open platform for visualizing, downloading, and analyzing multidimensional cancer genomics and clinical data (33). Here, we selected the “TCGA pancancer atlas studies” covering 10,967 samples across 32 cancer types to further explore *FGFR2* alterations among different cancers (Supplementary Table 1). The *FGFR2* mRNA expression data downloaded from cBioportal was generated from normalized values with the reference population of all samples independent of sample diploid status, termed as NormalizeExpressionLevels _allsampleref.py, and was log10 transformed. For the *FGFR2* CNV data, the log ratio value represents: 2 = amplification; 1 = gain; 0 = diploid; −1 = shallow deletion; and −2 = deep deletion.

The clinical data for analyzing the association between *FGFR2* alteration and patient survival was downloaded from cBioportal; the association between *FGFR2* expression and patient survival was analyzed using the Kaplan-Meier Plotter (34), which is an online tool for exploring patient survival in different cancer types based on large sample datasets. Forest plots were drawn to summarize the survival analysis. The 95% confidence interval (CI), hazard ratio (HR), and *p*-values were collected.

### Statistical Analyses

The statistical analyses were analyzed by Student's *t*-test, Cox regression analysis, and linear regression analysis when appropriate. *p* < 0.05 was set as statistically significant if there was no special annotation. Online analysis websites of GEPIA2 (http://gepia.cancer-pku.cn/), cBioportal (http://cbioportal.org/), GSCALite (http://bioinfo.life.hust.edu.cn/web/GSCALite/), and Kaplan-Meier Plotter (http://kmplot.com/) were also used.

## Results

### Pancancer Expression and Methylation Analysis of *FGFR2*

**Figure 1:**
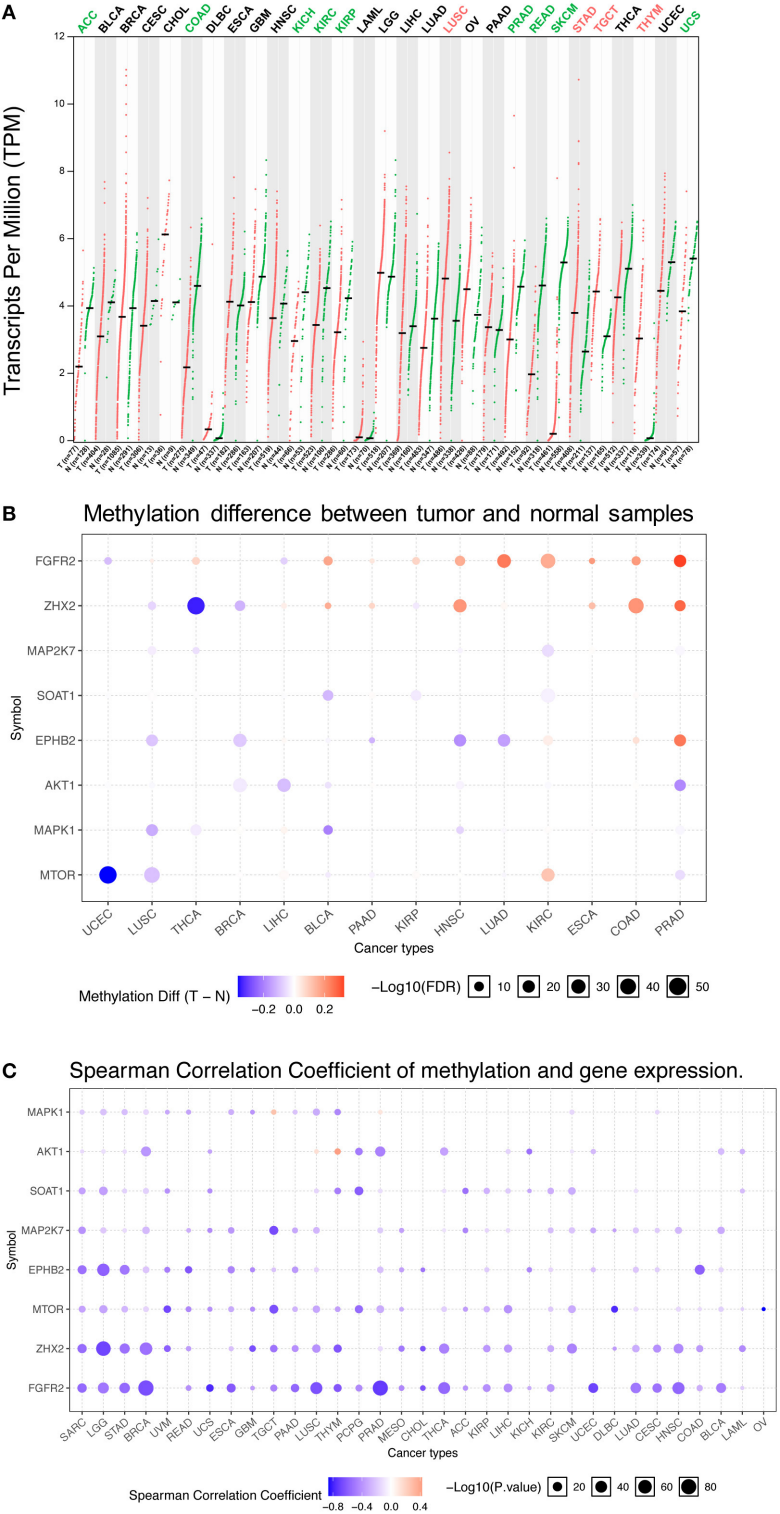
*FGFR2* mRNA expression and methylation in TCGA cancer tissues. **(A)** *FGFR2* mRNA expression between tumors and the corresponding normal tissues across TCGA cancer types from GEPIA. **(B)** Bubble map of the differential methylation of *FGFR2* and downstream genes between tumors and the corresponding normal tissues in 14 cancer types. Red dots indicate increased methylation in cancers, and blue dots indicate decreased methylation in cancers. **(C)** Bubble map showing the association between methylation and the expression of *FGFR2* and downstream genes across different cancer types. Red dots represent the upregulated methylation level and expression level, and blue dots represent the upregulated methylation level and downregulated expression level. For the point in two bubble map: the size of the point represents statistical significance, where the larger the size, the greater the significance. The color depth of the point represents the difference, where the darker the color, the greater the difference. TPM, transcripts per million.

Abnormal *FGFR2* expression has been reported in various cancer types (35–37). Previous studies on *FGFR2* expression in cancer have used several different research methods and are limited to small sample sizes and/or to single or limited numbers of cancer types. In this study, a more comprehensive analysis of *FGFR2* expression in pancancer was provided. First, we compared the mRNA expression profiles of *FGFR2* between tumors and the corresponding normal tissues of various cancer types by GEPIA2 (Figure 1A). The TCGA and GTEx gene expression data of *FGFR2* were re-computed from raw RNA-Seq data by the UCSC Xena project based on a standard pipeline to make data from these two sources more compatible (29, 30). Compared with the corresponding normal tissue, significantly differential expression was found in 13 cancer types, with four cancer types upregulated (lung squamous cell carcinoma (LUSC), stomach adenocarcinoma (STAD), testicular germ cell tumors (TGCT), and thymoma (THYM)) and nine cancer types downregulated (adrenocortical carcinoma (ACC), colon adenocarcinoma (COAD), kidney chromophobe (KICH), kidney renal clear cell carcinoma (KIRC), kidney renal papillary cell carcinoma (KIRP), prostate adenocarcinoma (PRAD), rectum adenocarcinoma (READ), skin cutaneous melanoma (SKCM), and uterine carcinosarcoma (UCS)) (Figure 1A). The cancer type with the most increased expression was THYM, with 3.0 TPM (tumor) compared with 0.07 TPM (normal tissue). The cancer type with the most decreased expression was SKCM, with 0.2 TPM (tumor) compared with 5.3 TPM (normal tissue).

Increasing studies have demonstrated that DNA methylation is strongly correlated with altered gene expression in cancers (38, 39). Thus, we evaluated the methylation profiles of *FGFR2* and its downstream genes in various TCGA cancers by using the GSCALite platform (1, 31) (Figures 1B,C). First, we explored the methylation difference between tumor and normal tissues in 14 cancer types. The result showed that the methylation of *FGFR2* was upregulated in most cancer types including LUSC, thyroid carcinoma (THCA), bladder urothelial carcinoma (BLCA), pancreatic adenocarcinoma (PAAD), KIRP, head and neck squamous cell carcinoma (HNSC), lung adenocarcinoma (LUAD), KIRC, esophageal carcinoma (ESCA), COAD, and PRAD (Figure 1B). Then, we evaluated the correlation between methylation and FGFR2 expression in 32 cancer types, the result showed that the expression levels of *FGFR2* and downstream genes were mainly negatively correlated with methylation, with only a few positive correlations (Figure 1C).

### *FGFR2* Alterations (Mutation and CNVs) in Different Cancer Types

**Figure 2:**
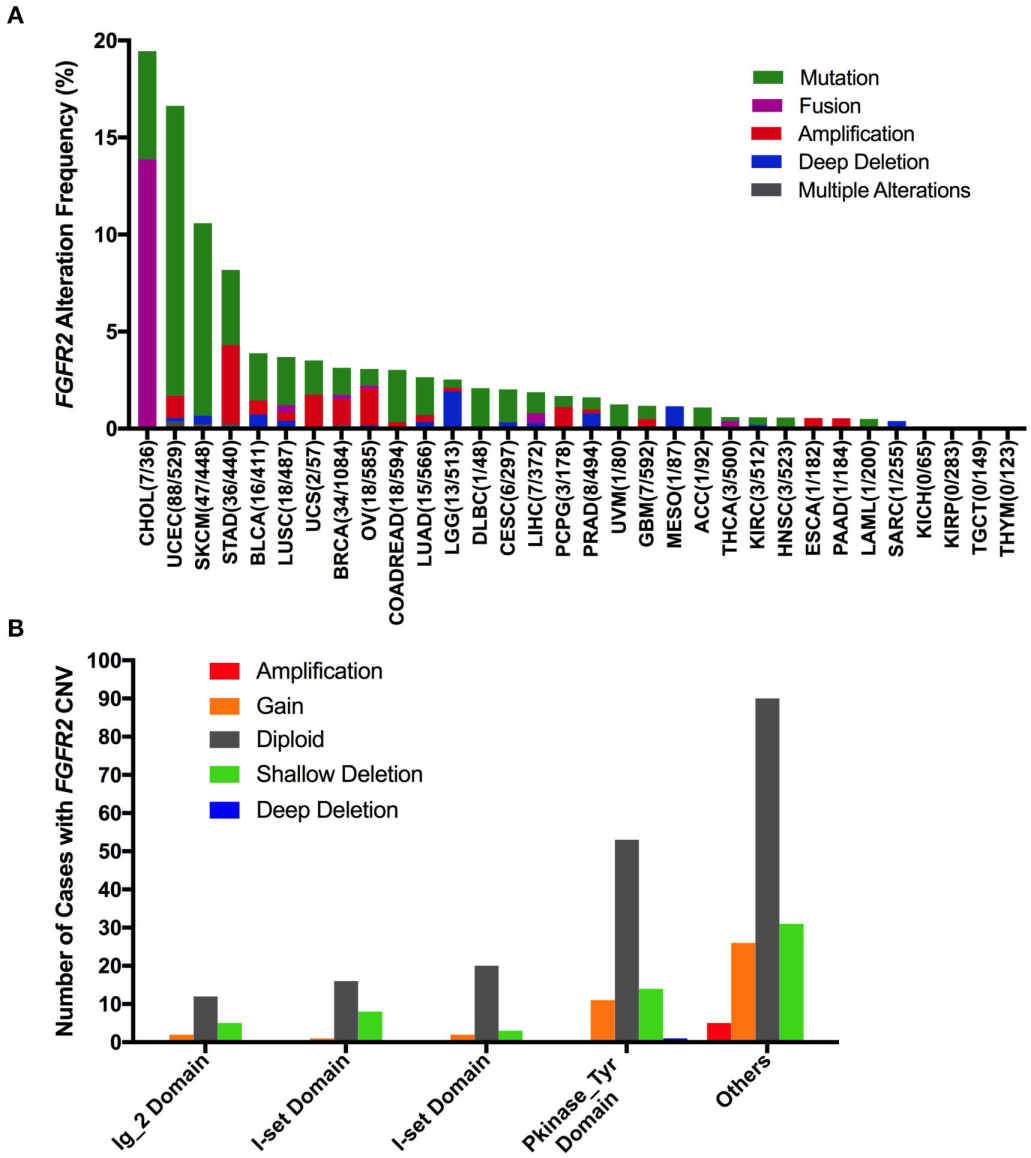
*FGFR2* alteration frequency and distribution across TCGA cancer types. **(A)** *FGFR2* alteration (mutation and CNVs) frequency in 32 cancer types. **(B)** The distribution of CNV types along with mutations located in different *FGFR2* functional domains. *FGFR2* functional domains: Ig_2 (43–115 aa), I-set (172–248 aa), I-set (264–359 aa), and PKinase_Tyr domains (481–757 aa). aa, amino acid.

The *FGFR2* alteration (mutation and CNV) frequency in all TCGA cancer types was ~3.3% (360 of 10,953 patients, 360 of 10,967 samples). However, the frequencies among different cancer types were quite variable (Figure 2A). *FGFR2* alterations were observed most commonly in CHOL (19.44%), in which fusion was more common (fusion, 13.88%; mutation, 5.56%). Other cancer types that also had *FGFR2* fusion but had much lower fusion rates included LUSC (0.41%), breast invasive carcinoma (BRCA) (0.28%), ovarian serous cystadenocarcinoma (OV) (0.17%), liver hepatocellular carcinoma (LIHC) (0.54%), and THCA (0.4%). Moreover, several cancer types mainly had *FGFR2* mutations but relatively few amplifications and/or deep deletions, such as UCEC, SKCM, BLCA, LUSC, COADREAD, LUAD, and so on (14.9 vs. 1.7%, 9.9 vs. 0.68%, 2.43 vs. 1.46%, 2.46 vs. 1.23%, 2.69 vs. 0.34%, and 1.94 vs. 0.7%, respectively). Tumors with dominant *FGFR2* amplification included STAD (4.09%), OV (1.88%), pheochromocytoma and paraganglioma (PCPG) (1.12%), ESCA (0.55%), and PAAD (0.54%). Deep deletion was more common in brain lower grade glioma (LGG), mesothelioma (MESO), and sarcoma (SARC) (1.95 vs. 0.58%, 1.15 vs. 0.0%, and 0.39 vs. 0.0%, respectively). Although the *FGFR2* alteration frequencies of the two lung cancer subtypes (LUSC and LUAD) were similar, LUSC had *FGFR2* fusion, but LUAD did not. Cancer types, including kidney chromophobe (KICH), KIRP, TGCT, and THYM, had neither *FGFR2* CNVs nor *FGFR2* mutations (Figure 2A).

Interestingly, mutation location and CNV occurrence were found to be correlated (Figure 2B). According to the Pfam database, FGFR2 harbors four functional domains, including the Ig_2 (43–115 aa), I-set (172–248 aa), I-set (264–359 aa), and PKinase_Tyr domains (481–757 aa). Here, we found 309 *FGFR2* somatic mutations across 32 cancer types. Nearly one-third of the mutations (26 of 79 mutations) in the Pkinase_Tyr domain were accompanied by *FGFR2* copy gain, shallow deletion and deep deletion, while ~40% of the mutations (62 of 152 mutations) in the other function-unknown domain had amplification, gain, and shallow deletion. Mutations in the Ig_2 domains and I-set domains rarely had concurrent CNVs (Figure 2B).

### *FGFR2* Somatic Mutation Patterns Across Cancer Types

**Figure 3:**
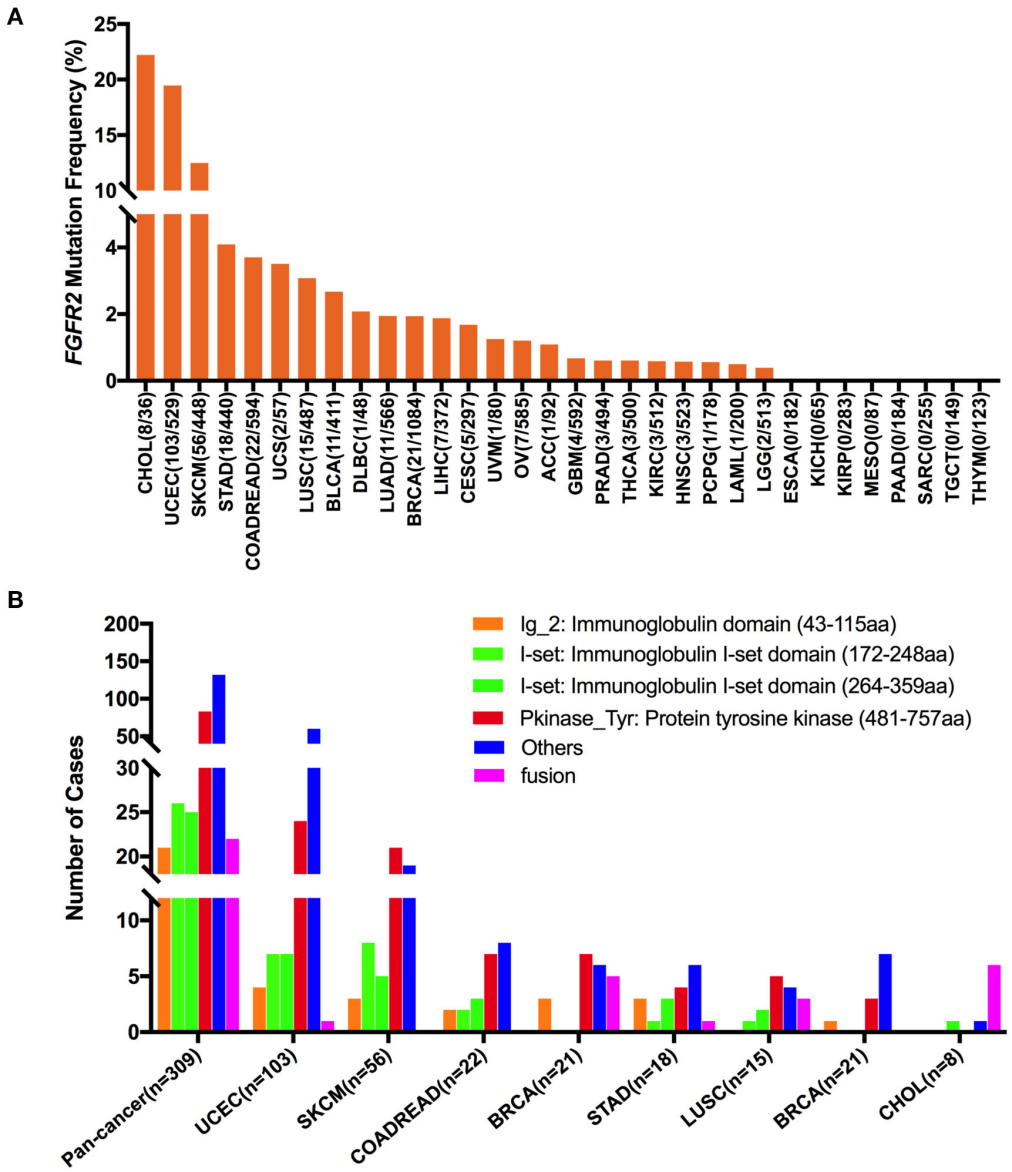
*FGFR2* mutation distribution in 32 TCGA provisional cancer types and protein functional domains. **(A)** *FGFR2* mutation frequency across different cancer types. **(B)** *FGFR2* mutation distribution in different functional domains in all cancer types and in the top 8 cancer types.

The overall mutation frequencies of *FGFR2* were 2.8% (309/10,967) for all tumor samples and 2.4% (262/10,953) for all patients across the 32 cancer types. The most common cancer types with *FGFR2* mutations were CHOL (12.3%), UCEC (10.5%), SKCM (8.8%), and STAD (4.2%). In contrast, ESCA, KICH, KIRP, MESO, PAAD, SARC, TGCT, and THYM showed almost no *FGFR2* mutations (Figure 3A). The total number of tumor samples from individual cancer types varied from 36 (CHOL) to 1,084 (BRCA), and those with too few tumor samples might not represent the complete picture of *FGFR2* mutation status (Supplementary Table 2).

Here, 309 *FGFR2* somatic mutations were observed across 32 cancer types and were broadly distributed across different functional domains of *FGFR2* (Figure 3B). The most common domains with *FGFR2* mutations were the other domain (132 samples) and the Pkinase_Tyr domain (83 samples), followed by the I-set domain (172–248 aa, 26 samples), the I-set domain (264–359 aa, 25 samples), and the Ig_2 domain (21 samples). Fusions (22 samples) were also observed in *FGFR2* somatic mutations. The location distribution of these *FGFR2* somatic mutations was quite different among different cancer types. Mutations in UCEC, COADREAD, STAD, and BLCA were most commonly located in the other domain, the functions of which were rarely known, especially for BLCA, which amounted to nearly three-fifths of all somatic mutations. Mutations in SKCM, BRCA, and LUSC were primarily in the Pkinase_Tyr domain. Meanwhile, fusion was found in UCEC, BRCA, STAD, LUSC, and CHOL, and especially in CHOL, fusion was the most common mutation, amounting to approximately three-fourths of all mutations (Figure 3B and Supplementary Table 3).

Based on their functional impact on protein coding, these 309 *FGFR2* somatic mutations could be classified into four categories: missense (254 mutations), truncating (29 mutations), fusion (22 samples), and in-frame (four mutations). S252 in the other domain was the most frequently mutated position, which was observed in 26 samples (all samples with S252W). Mutations at this position almost exclusively occurred in UCEC samples (24/26). S252W is known to be oncogenic and may be targetable with selective FGFR-targeted inhibitors (26, 40). The other tumors with mutations at this position were UCS (one sample) and OV (one sample). The second most mutated position was N549 in the Pkinase_Tyr domain, which was observed in 16 samples (12 samples with N549K (seven UCEC, three BRCA, one BLCA, one LUAD), two samples with N549H (two UCEC), two samples with N549D (two UCEC)) (Supplementary Figure 1). N549K and N549H are known to be oncogenic and likely oncogenic, respectively; however, the oncogenic function of N549D is unknown, and there are no Food and Drug Administration (FDA)-approved treatments specifically for patients with mutations at this position.

**Figure 4:**
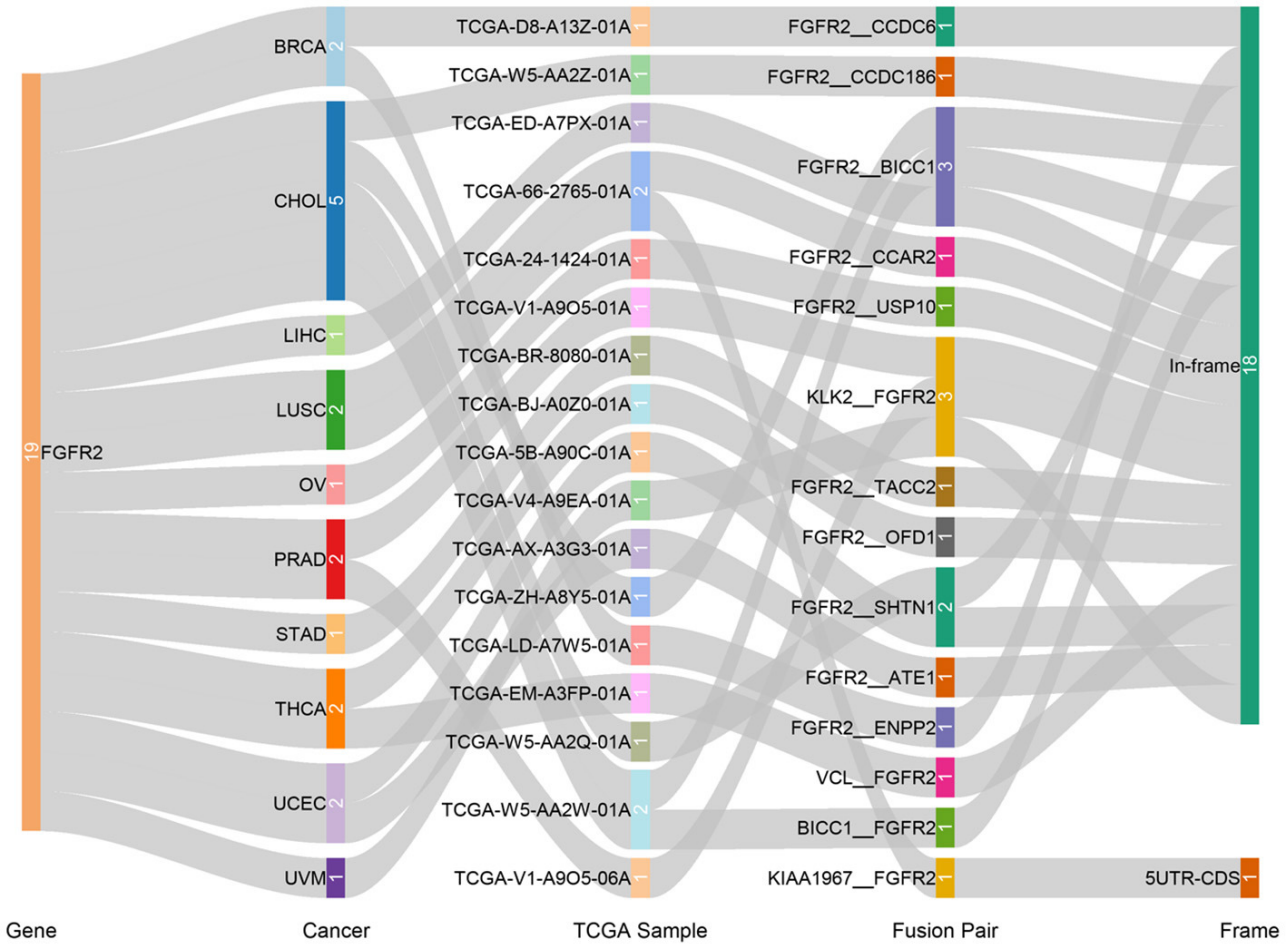
Fusion gene of *FGFR2* in TCGA cancer types.

Fusion genes produced by genomic-level cleavage and resplicing are often targets for tumor diagnostic treatment. Based on the TCGA Fusion Gene Database (32), we detected fusion transcripts of *FGFR2* across different cancer types with high confidence (Figure 4). *FGFR2* fusion transcripts were detected in CHOL (5), BRCA (2), LUSC (2), PRAD (2), THCA (2), UCEC (2), LIHC (1), OV (1), STAD (1), and uveal melanoma (UVM) (1). CHOL harbored the highest number of fusion transcripts (two *FGFR2*__*BICC1*, one *BICC1*__*FGFR2*, one *FGFR2*__*SHTN1*, one *FGFR2*__*CCDC186*). The only other tumor with the *FGFR2*__*BICC1* fusion transcript was LIHC (one *FGFR2*__*BICC1*). Most of these *FGFR2* fusion transcripts belonged to the in-frame class, while *KIAA1967*_*FGFR2* in LUSC belonged to the 5′ UTR-CDS class.

**Figure 5:**
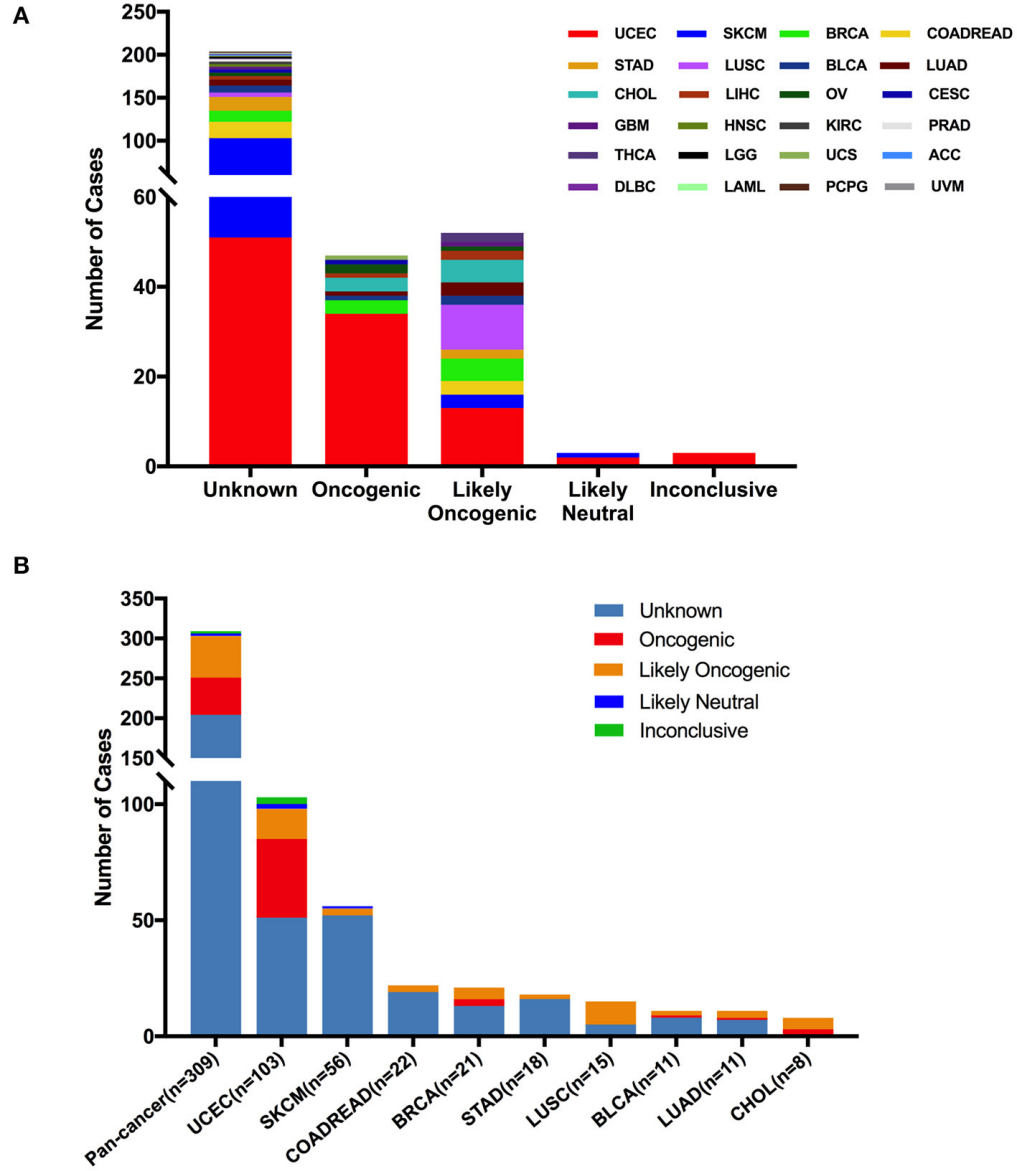
*FGFR2* mutation distribution based on functional impacts. **(A)** *FGFR2* mutation classification by functional impacts on all cancer types together. **(B)** Functional impact class distribution of *FGFR2* mutations in all and top 9 cancer types.

According to oncogenic effects and predictive significance (41), the 309 *FGFR2* mutations could be classified into five categories. As shown in Figure 5, oncogenic (47 mutations), likely oncogenic (52 mutations), likely neutral (three mutations), inconclusive (three mutations), and unknown (204 mutations). Approximately half of these somatic mutations belonged to the unknown class, highlighting the challenge of interpreting the meanings of these mutations. However, in LUSC and CHOL, most mutations were mainly distributed in the functional categories. Meanwhile, nearly half of *FGFR2* mutations in UCEC were also distributed in the functional classes (34 oncogenic, 13 likely oncogenic) (Figure 5 and Supplementary Table 2).

**Figure 6:**
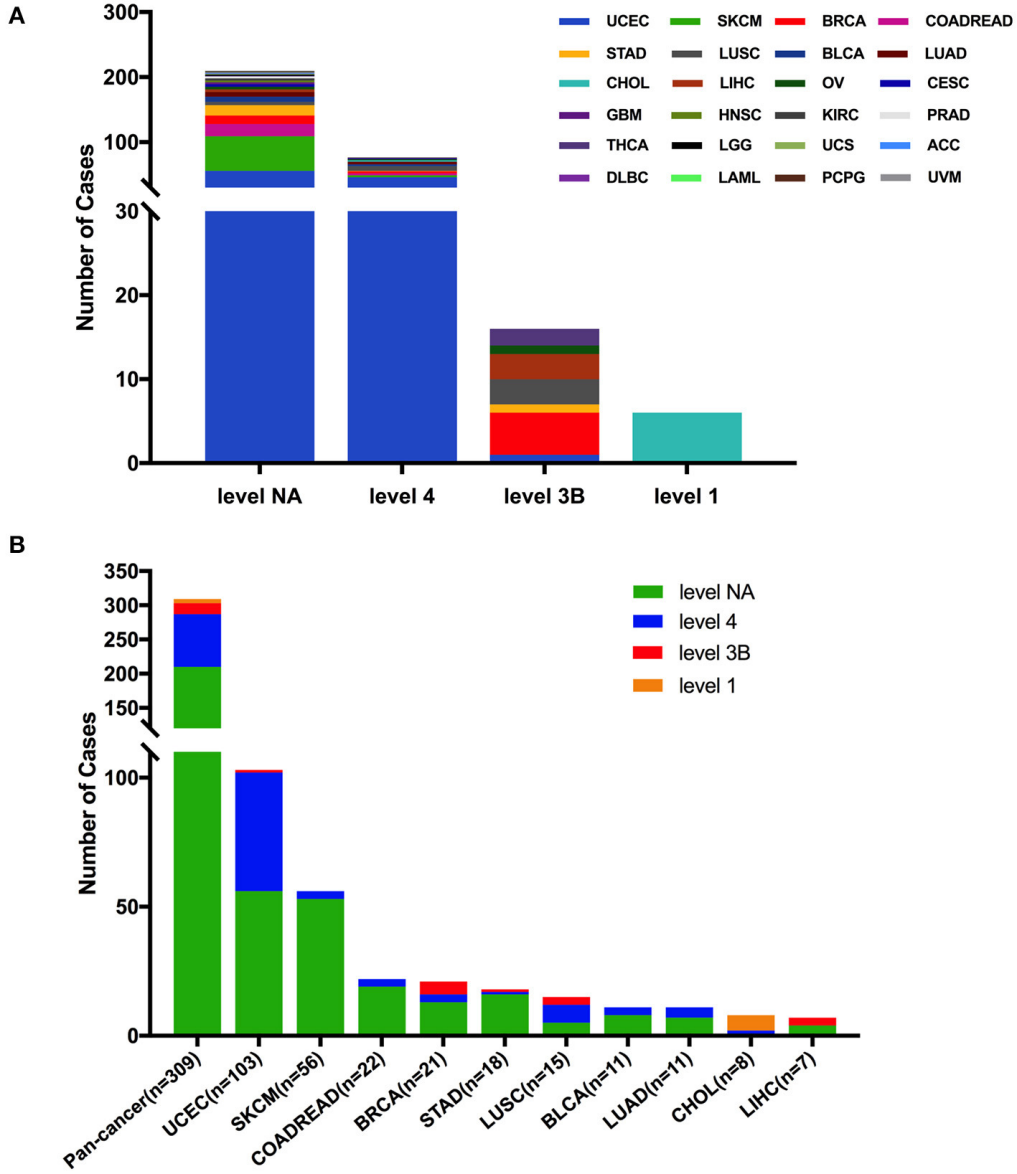
*FGFR2* mutation classification based on targeted therapy implications. **(A)** *FGFR2* mutation classification based on the clinical targeted treatment implications as annotated in OncoKB across 32 TCGA cancer types together. **(B)** Targeted treatment implications distribution of *FGFR2* mutations for all TCGA cancers together and for top 9 cancer types.

Based on the clinical targeted therapy implications defined using OncoKB from cBioPortal (33), these 309 *FGFR2* somatic mutations could be divided into four levels: level 1 (6 mutations), level 3B (16 mutations), level 4 (77 mutations), and level NA (210 mutations) (Figure 6). Most of them were in the NA class, suggesting that more efforts are needed to explore targeted therapy. Only level 1 was represented for targeted therapy with an FDA-approved drug (42). All level 1 mutations were observed in CHOL, which were all fusions. In UCEC, although oncogenic/likely oncogenic mutations accounted for nearly half of the mutation samples (47 of 103 mutations), most of them were in the level NA and level 4 categories without targeted therapy implications (Figure 6 and Supplementary Table 2).

### *FGFR2* CNVs in Different Cancer Types

**Figure 7:**
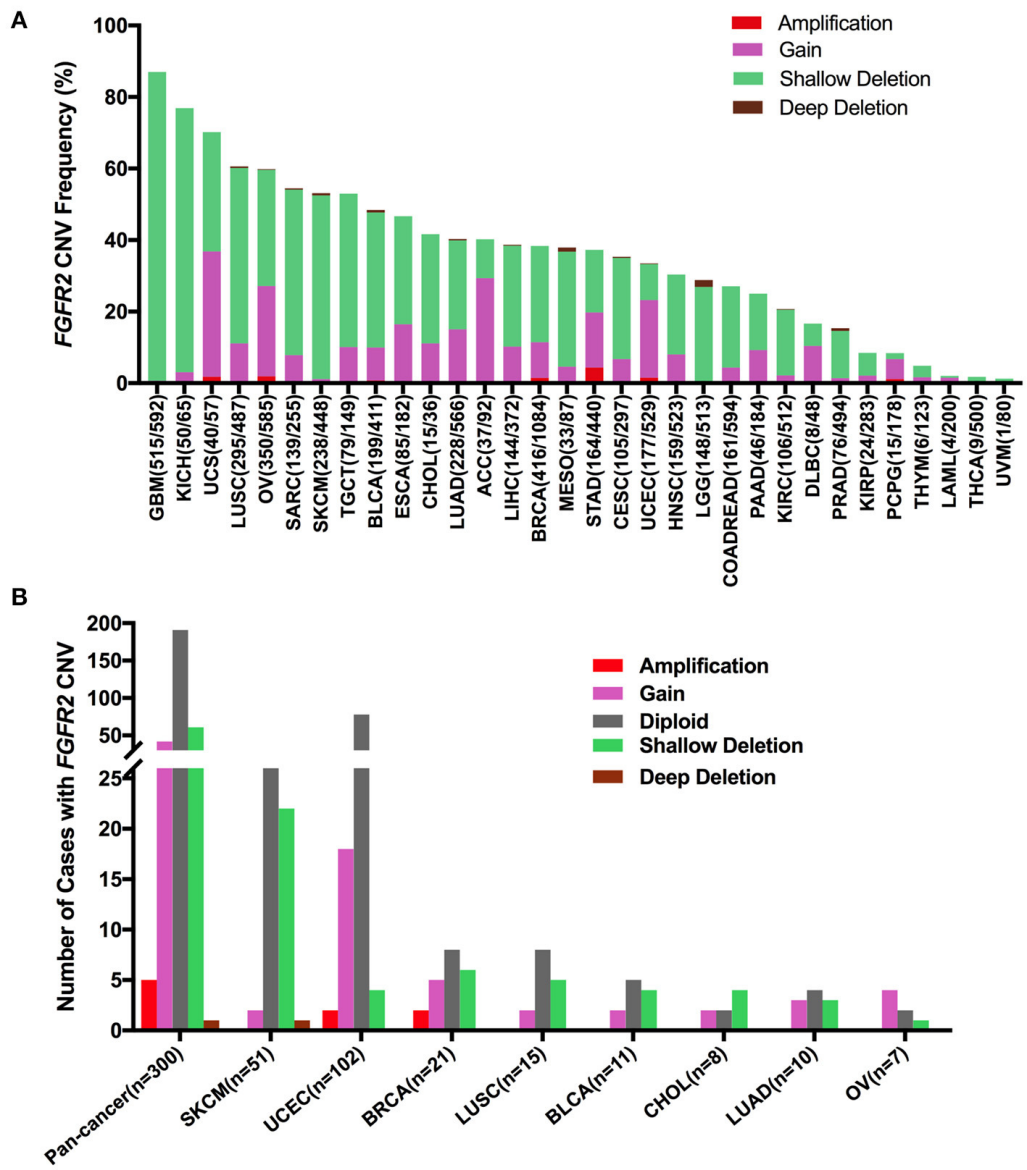
*FGFR2* CNV distribution across TCGA cancer types. **(A)** *FGFR2* CNV frequency across 32 TCGA cancer types. **(B)** *FGFR2* CNV distribution in all and top 8 cancer types for the cases with *FGFR2* mutations simultaneously. CNV, copy number variant.

First, *FGFR2* mRNA expression was compared across all TCGA cancer types and exhibited a broad spectrum (Supplementary Figure 2A), indicating that a specific cancer type may have unique genetic features that drive *FGFR2* expression. Meanwhile, according to the interquartile range, the spread of *FGFR2* expression varied in several tumor types more than others. For example, COADREAD and BRCA had a widespread distribution, while TCGT had a narrow spread of *FGFR2* expression, which may be due to some cancer types harboring more than one subtype and therefore having more genetic diversity. Next, we analyzed *FGFR2* CNVs across cancer types using ciBioportal (Figure 7). The overall *FGFR2* CNV frequency was ~37.1% (4,072 of 10,967 samples). Most of the CNVs were shallow deletions (3,577 samples), followed by gain (924 samples), amplification (73 samples), and deep deletion (31 samples). The most common tumors with *FGFR2* CNVs were glioblastoma multiforme (GBM) (87.0%), KICH (76.9%), UCS (70.2%), LUSC (60.6%), and OV (60.0%). In contrast, acute myeloid leukemia (LAML) (2.0%), THCA (1.8%), and UVM (1.25%) exhibited very low frequencies of *FGFR2* CNVs (Figure 7A). Then, we wanted to determine whether *FGFR2* CNVs were correlated with *FGFR2* expression. *FGFR2* CNVs with *FGFR2* mRNA expression were compared across all TCGA tumor types. The correlation analysis showed that there was a positive correlation between *FGFR2* CNVs and mRNA expression in pancancer (*r* = 0.1578, *p* < 0.0001) (Supplementary Figure 2B). In addition, some other factors might also affect the expression of *FGFR2*, such as methylation (43).

Furthermore, we found that among the 309 samples with *FGFR2* somatic mutations described above, 109 also harbored *FGFR2* CNVs, of which 42 harbored gains, five harbored amplifications, 61 harbored shallow deletions, and one harbored a deep deletion. SKCM had the highest number of shallow deletions, and UCEC had the highest number of gains (Figure 7). As shown in Figure 7A and Supplementary Figure 2A, SKCM had a very high proportion of shallow deletions and had lower *FGFR2* expression. Similarly, UCEC had a relatively high proportion of amplification/gain and had higher *FGFR2* expression. However, GBM and KICH had the highest proportions of shallow deletions, in which *FGFR2* expression was not that low, indicating that additional genetic alterations may contribute to the expression of *FGFR2* in these cancer types.

### *FGFR2* Alterations and Patient Survival

**Figure 8:**
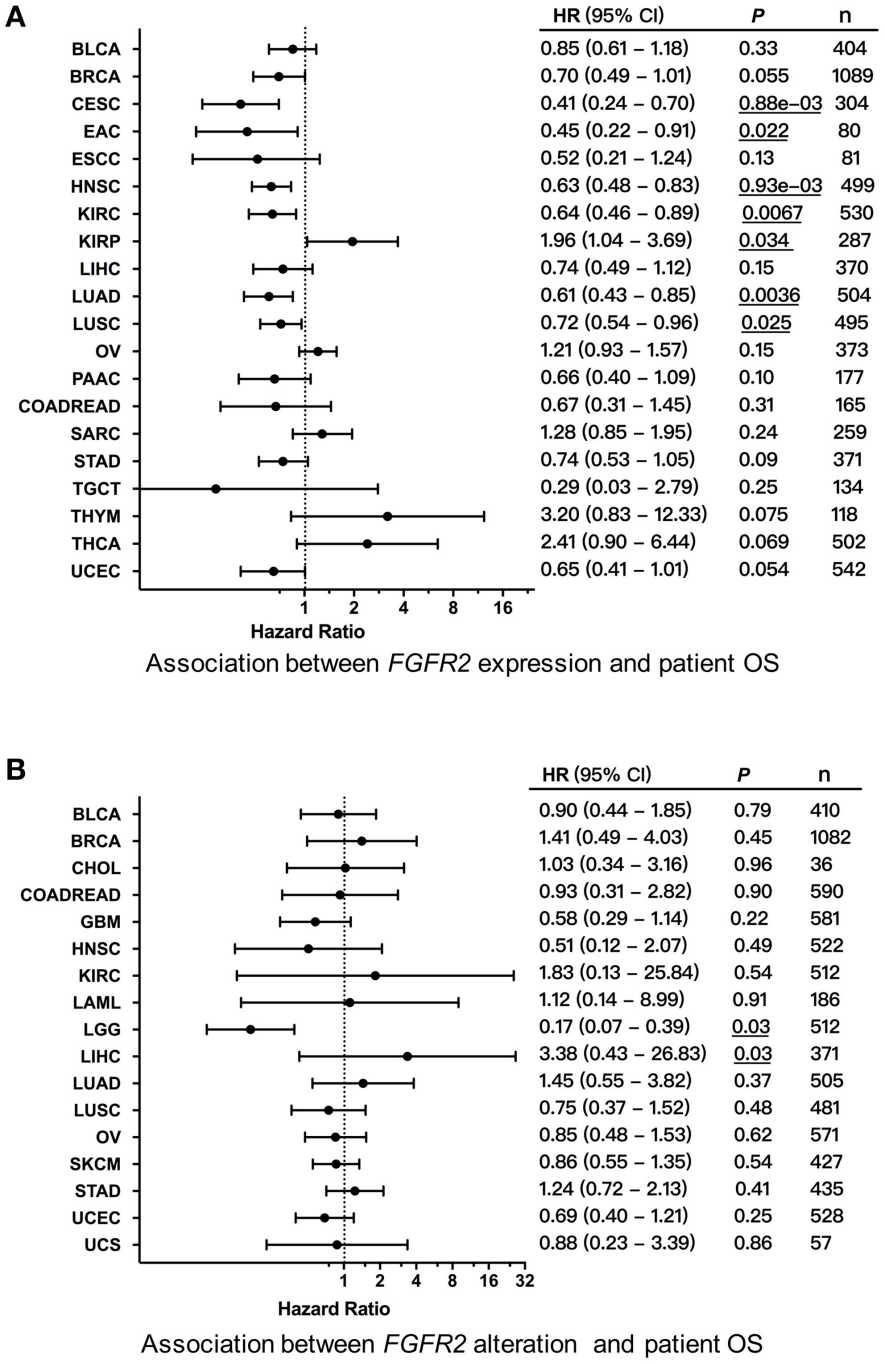
Association between *FGFR2* alterations and patient survival. **(A)** Forest plot for the association between *FGFR2* expression and patient overall survival (OS). **(B)** Forest plot for the association between *FGFR2* alterations and patient OS. These significant *P*-values are underlined.

We first explored the clinical significance of *FGFR2* expression, and the association between *FGFR2* mRNA expression and patient overall survival (OS) and progression-free survival (PFS) in individual cancer types was analyzed. We found that decreased *FGFR2* expression was associated with short patient OS in cervical squamous cell carcinoma and endocervical adenocarcinoma (CESC), EAC, HNSC, KIRC, LUAD, and LUSC, while increased *FGFR2* expression was associated with short patient OS in KIRP (Figure 8A). In addition, survival association analysis between *FGFR2* mRNA expression and patient RFS in each cancer type showed that among patients with LIHC or THCA, decreased *FGFR2* expression was associated with short patient RFS, while among patients with BLCA or PCPG, increased *FGFR2* expression was associated with short RFS (Supplementary Figure 3). Moreover, to further explored the clinical significance of *FGFR2* alteration, we analyzed survival association regarding alteration status in individual cancer types. The results showed that *FGFR2* alteration was associated with a better prognosis in LGG, which *FGFR2* alteration was associated with short patient OS in LICH (Figure 8B).

## Discussion

In our study, the *FGFR2* profiles were mainly analyzed by the cBioportal approach, which would unify the TCGA data across different cancer types with uniform clinical elements and ideally processed curation. We profiled various cancer types and provided significant and comprehensive information regarding *FGFR2* abnormal expression, methylation, mutation, CNVs, and alteration that differed greatly across different cancers, which had critical therapeutic and prognostic implications. The large comprehensive datasets evaluated here covered over 10,000 tumor samples across more than 30 cancer types. The total alteration frequency of *FGFR2* across all tumor types was relative low (~0–20%), and mutation took up a major portion in most cancer types. Targetable mutations were mainly observed in CHOL and could be targeted by pemigatinib, a selective competitive inhibitor of FGFR1, FGFR2, and FGFR3 approved by FDA recently. Genetic fusions were mainly detected in CHOL, followed by BRCA, LUSC, PRAD, THCA, and UCEC. *BICC1* was found to be the most common partner gene of *FGFR2*. CHOL, UCEC, and SKCM are the three cancer types that harbor the highest frequencies of *FGFR2* alteration. In CHOL, fusion accounted for the major proportion, while in UCEC and SKCM, mutation was the dominant alteration. Mutations in these two cancer types were most commonly located in the Pkinase_Tyr domain and the other function-unknown domain. S252W, the most common mutation in UCEC, is located in another domain of unknown function, and its role in targeted therapy implications is not well known. In addition, CHOL and UCEC had high *FGFR2* expression, and SKCM had low *FGFR2* expression, but in these cancer types, there was no survival association shown between *FGFR2* expression and patient prognosis. In addition, other cancer types, including KICH, KIRP, TGCT, and THYM, had almost no *FGFR2* alterations. Moreover, some common tumors with *FGFR2* alterations, including BLCA, LUSC, COADREAD, and LUAD, had similar characteristics: an alteration frequency of ~2–4% and mutation as the most common type. Over half of the mutations in LUSC belonged to the likely oncogenic class and were level 3B and level 4. Conversely, certain cancer types, including OV, PCPG, ESCA, and PAAD, mainly harbor *FGFR2* amplification but rarely mutations. Meanwhile, deep deletion was the dominant alteration type in LGG and MESO; however, in LGG, high expression of *FGFR2* was observed, indicating that additional genetic alterations may contribute to high expression of *FGFR2* in this cancer type.

A previous study reported that *FGFR2* fusions are observed mostly in cholangiocarcinoma, occurring in 10–16% of patients (24, 44, 45). In our study, we found that cholangiocarcinoma had the highest frequency of *FGFR2* alteration, and fusion accounted for the major proportion. Fusion types in CHOL included *FGFR2*__*BICC1, BICC1*__*FGFR2, FGFR2*__*SHTN1*, and *FGFR2*__*CCDC186*. The first two fusion types are known to be oncogenic, and the last two are likely oncogenic. Consistent with previous findings (46), in our analysis, *BICC1* was found to be the most common partner gene of *FGFR2*. However, different fusion types did not seem to exhibit different effects on the therapeutic and prognostic implications of patients (47–50). According to the targeted therapy implications defined by OncoKB, all these fusions in CHOL were classified into level 1, which represented the treatment of patients with an FDA-approved drug. In April 2020, pemigatinib, a selective competitive inhibitor of FGFR1, FGFR2, and FGFR3, was approved in the USA for the targeted therapy of patients with previously treated, locally advanced or metastatic CHOL and *FGFR2* fusions (24, 25). Pemigatinib was the first targeted therapy for CHOL in the USA. Meanwhile, in some other FGFR-driven malignant tumors, such as bladder urothelial carcinoma, the treatment implications of pemigatinib have also been explored in the clinic across various countries (51, 52). Other drugs are also undergoing clinical development for use in CHOL or other solid tumors. BGJ398, a selective pan-FGFR-targeted inhibitor, has shown meaningful clinical activity against chemotherapy-refractory CHOL with *FGFR2* fusions (27). Debio 1347 and erdafitinib, which are also pan-FGFR-targeted inhibitors, have exhibited preliminary clinical activity in BRCA/CHOL and BLCA/CHOL, respectively (26, 28).

Frequent activating *FGFR2* mutations have been observed in ~12% of UCEC, suggesting an opportunity for targeted therapy (53, 54). In this study, we found that UCEC had the second highest frequency of *FGFR2* alteration, which was driven by a high proportion of mutations. The most frequently mutated positions in UCEC were S252W and N549K, and these two mutations are known to be oncogenic; however, they all belong to the level 4 class, which represents rare targeted therapy implications. Recently, several laboratory findings showed the potential implications of FGFR inhibitors in targeted therapy in UCEC (55, 56). For example, AZD4547, a selective pan-FGFR inhibitor, has shown therapeutic activity in a UCEC cell model (54). Additionally, FGFR inhibitors combined with PI3K inhibitors could enhance antitumor responses in *FGFR2*-mutant UCEC (57). However, clinical trials based on FGFR mutation as a therapeutic target of FGFR inhibitors have exhibited disappointing clinical outcomes, and there is still no FDA-approved drug for the clinical targeted therapy of UCEC, suggesting that more efforts are needed to test the efficacy of FGFR inhibitors to prevent the progression and recurrence in UCEC. Some reports have indicated that *FGFR2* mutations are associated with poor prognosis in patients with UCEC (53). However, in this large TCGA dataset, there was no survival association observed between *FGFR2* alterations and patient prognosis. This paradox could be due to insufficient sample size; more research needs to be conducted on this aspect.

LUSC and LUAD are two lung cancer subtypes. *FGFR2* expression in these two cancer types was associated with patient OS. Their *FGFR2* alteration frequencies were ~3% and had similar alteration patterns, in which mutation was the most common alteration. However, LUSC featured *FGFR2* fusion, which was not present in LUAD. The fusions in LUSC were divided into level 3B, and the remaining mutations were in level 4/level NA classes without targeted therapy implications. BGJ398, a pan-FGFR inhibitor, showed antitumor activity in LUSC (40) and is expected to be approved to treat LUSC by the FDA in the future. In addition, over half of the mutations in LUSC were in the likely oncogenic class, and high expression of *FGFR2* was found in LUSC tissues compared with corresponding normal tissues. However, most mutations in LUAD were in the unknown class, suggesting that more efforts are needed to further explore the roles of these unknown mutations, and they may also have critical functional roles in driving oncogenesis.

In this study, we profiled 32 cancer types regarding *FGFR2* expression, methylation, alteration and their clinical associations. However, there were several limitations that need to be mentioned. First, even though this was a pancancer global analysis, it lacked an in-depth analysis of individual tumor types. Additionally, several rare tumor types did not harbor sufficient sample sizes, which made the full expression, methylation and alteration spectrum difficult to capture. Furthermore, the frequency of *FGFR2* alteration across all tumor types was between 0 and 20%, and this low alteration frequency could make our analysis challenging. Furthermore, even though the TCGA and GTEx gene expression data of *FGFR2* were re-computed from raw RNA-Seq data by the UCSC Xena project based on a standard pipeline to minimize differences from these two sources (29, 30), minimization is not the elimination of the problem, the differences between these two datasets still exist. The FGFR2 expression analysis obtained here needed to be validated with more samples for each individual cancer types in the future. Finally, for analyzing the FGFR2 methylation difference between tumor and normal tissues, the methylation profiles were obtained in only 14 cancer types instead of all 32 cancer types from the GSCALite web server, and there were still 18 tumor types lacked the methylation profiles. More efforts are needed to explore the methylation profiles in these 18 cancer types using more samples in the future, and in these cancers, FGFR2 methylation may also have critical functional roles on oncogenesis. Some critical leads exhibited from this analysis will provide guidance for future exploration.

## Conclusions

In conclusion, our study provided the first comprehensive pancancer view of *FGFR2* abnormal expression, methylation, alteration, and their therapeutic and prognostic implications, covering 10,967 tumor samples across 32 cancer types. While several tumors with low alteration frequency are correlated with prognosis, others with high alteration frequency were not. Some alterations are found to be more involved in oncogenesis, while other alternations are found to participate more in targeted therapy. Genomic profiling may offer directions for their application in clinical targeted therapy.

## Data Availability Statement

The original contributions presented in the study are included in the article/Supplementary Material, further inquiries can be directed to the corresponding authors.

## Author Contributions

YY and ZX: conception and design. JL, KH, and JH: writing, review, and/or revision of the manuscript. LZ: administrative, technical, or material support. All authors approved final version of manuscript.

## Conflict of Interest

The authors declare that the research was conducted in the absence of any commercial or financial relationships that could be construed as a potential conflict of interest.

## Supplementary Material

Supplementary Figure 1 *FGFR2* mutation distribution in different protein functional domains. **(A)** *FGFR2* mutation distribution in different protein functional domains of *FGFR2* across 32 TCGA cancer types together. **(B)** *FGFR2* mutation distribution in different protein functional domains of *FGFR2* in UCEC.

Supplementary Figure 2 *FGFR2* mRNA expression and its correlation with *FGFR2* CNVs. **(A)** *FGFR2* mRNA expression (RNA-seqV2 RSEM, log10 transformed) in all TCGA cancer types. **(B)** *FGFR2* linear copy number value correlations with *FGFR2* mRNA expression (RNA-seqV2 RSEM, log10 transformed) in different cancer types.

Supplementary Figure 3 Forest plot for the association between *FGFR2* expression and patient progression-free survival (PFS).

Supplementary Table 1 Summary of TCGA cancer types and sample size.

Supplementary Table 2 Detailed information on 309 human *FGFR2* somatic mutations from cBioportal for 32 TCGA cancer types.

Supplementary Table 3 Somatic mutation distribution in different functional domains of *FGFR2* for cancers with *FGFR2* mutations and all cancers together.
