# Supplementary figures and images for: A Pancancer Analysis of the Expression Landscape and Clinical Relevance of Fibroblast Growth Factor Receptor 2 in Human Cancers
Source: Front Oncol. 2021 Apr 21;11:644854. doi: 10.3389/fonc.2021.644854 (PMC8097147; doi:10.3389/fonc.2021.644854)

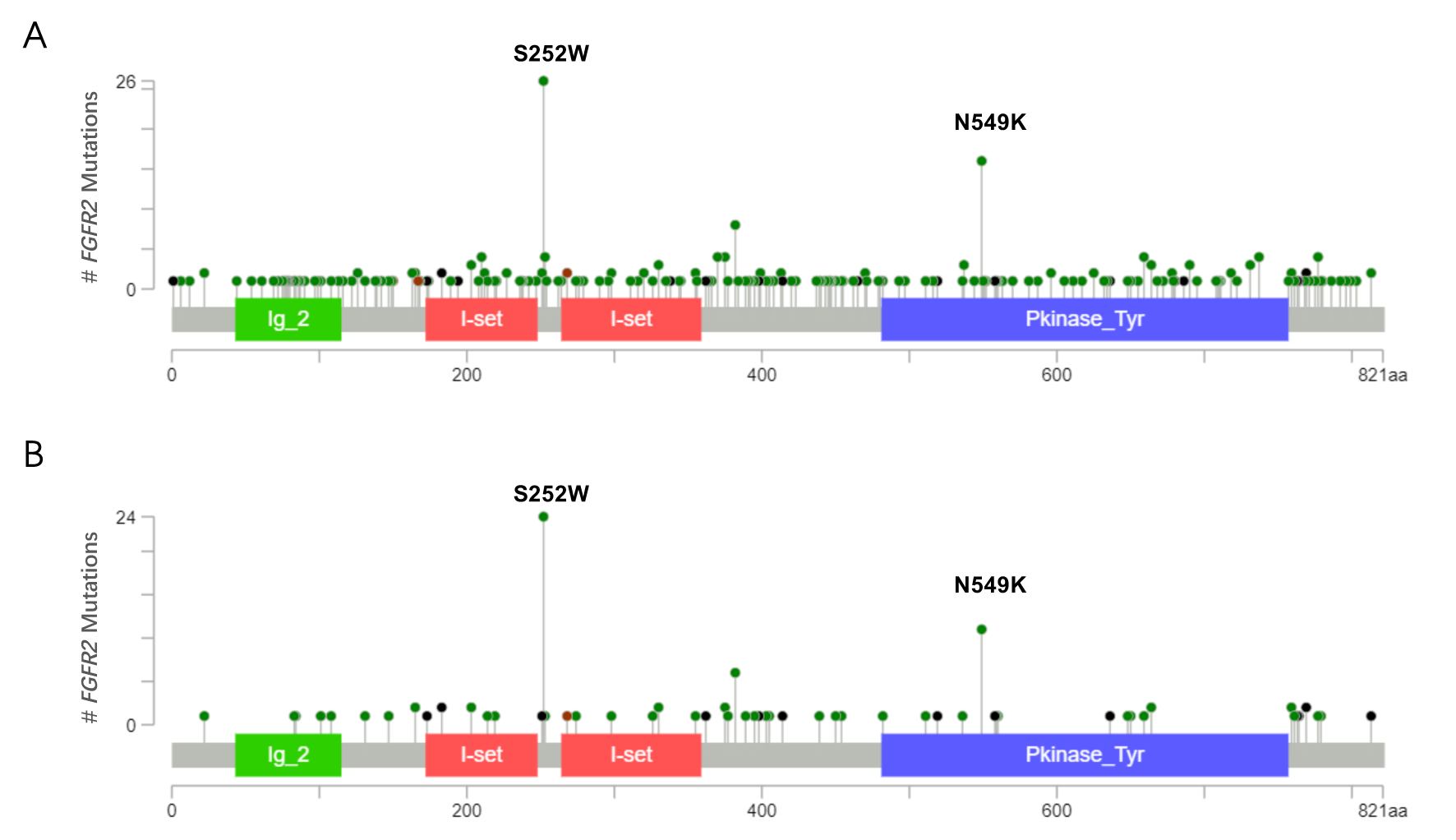

Supplement: Supplementary Figure 1 — FGFR2 mutation distribution in different protein functional domains. (A) FGFR2 mutation distribution in different protein functional domains of FGFR2 across 32 TCGA cancer types together. (B) FGFR2 mutation distribution in different protein functional domains of FGFR2 in UCEC. [file Image_1.TIF]

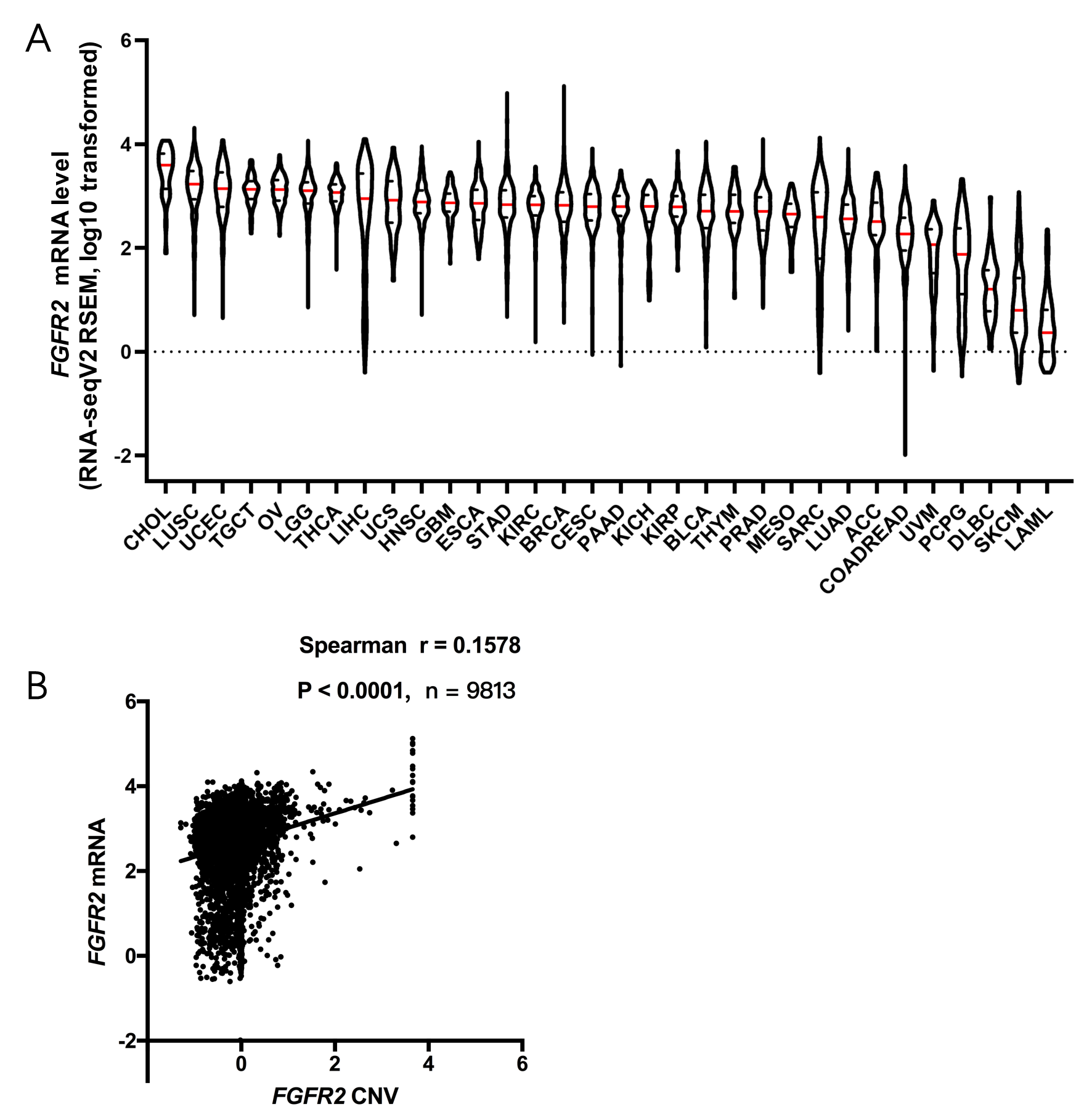

Supplement: Supplementary Figure 2 — FGFR2 mRNA expression and its correlation with FGFR2 CNVs. (A) FGFR2 mRNA expression (RNA-seqV2 RSEM, log10 transformed) in all TCGA cancer types. (B) FGFR2 linear copy number value correlations with FGFR2 mRNA expression (RNA-seqV2 RSEM, log10 transformed) in different cancer types. [file Image_2.TIF]

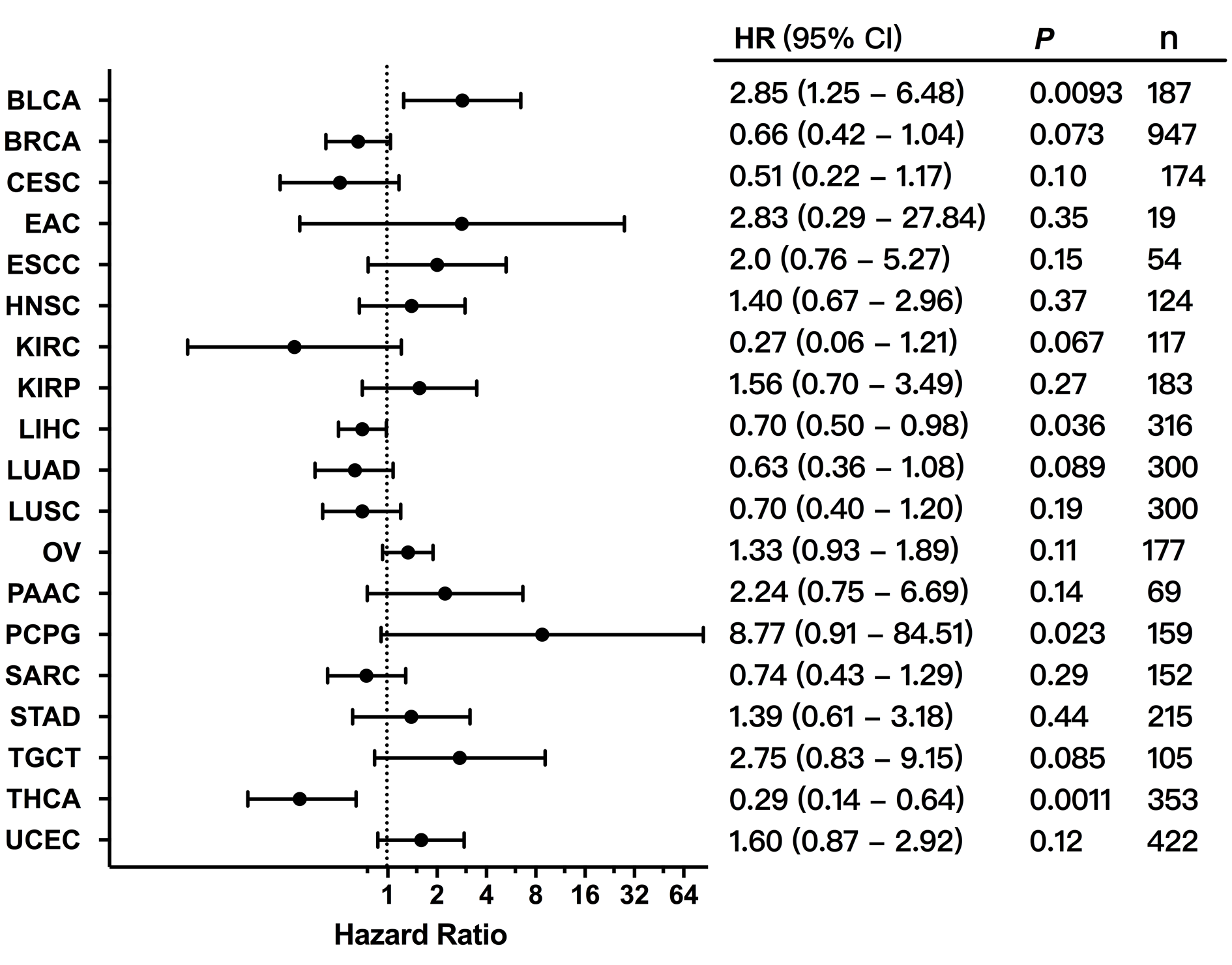

Supplement: Supplementary Figure 3 — Forest plot for the association between FGFR2 expression and patient progression-free survival (PFS). [file Image_3.TIF]
